# Full-length transcriptome provides insights into the molecular regulation of seed spike number in *Agropyron mongolicum*

**DOI:** 10.3389/fpls.2025.1570213

**Published:** 2025-06-03

**Authors:** Meiying Guo, Yaqin Wei, Xiqiang Liu, Xintian Huang, Yaling Liu, Yan Zhao

**Affiliations:** ^1^ College of Grassland Science, Inner Mongolia Agricultural University, Key Laboratory of Grassland Resources (IMAU), Ministry of Education, Key Laboratory of Forage Cultivation, Processing and High Efficient Utilization, Ministry of Agriculture, Hohhot, Inner Mongolia, China; ^2^ Institute of Ecological Protection and Restoration, Chinese Academy of Forestry/Grassland Research Center, National Forestry and Grassland Administration, Beijing, China; ^3^ Research Department of Inner Mongolia Grassland Technology Innovation Center Co., Ltd, Hohhot, Inner Mongolia, China

**Keywords:** *Agropyron mongolicum*, full-length transcriptome, spikes, gene, quantitative realtime PCR

## Abstract

*Agropyron mongolicum* is a xerophytic perennial grass with good palatability and nutritional value, predominantly distributed in sandy, arid regions and desert steppes of northwest China. However, its low biomass and limited seed yield pose significant challenges for large-scale cultivation and forage production. To date, there have been limited genomic or transcriptomic studies on *A. mongolicum*, particularly regarding the molecular genetic mechanisms underlying yield-related traits. In this study, we employed an integrated transcriptomic approach combining long-read sequencing (PacBio Iso-Seq) and short-read sequencing (Illumina RNA-seq) to generate a comprehensive full-length transcriptome from four tissues—leaves, stems, young spikes, and mature spikes. Transcriptomic comparisons between multi-spike and few-spike germplasms were performed to identify candidate genes involved in the regulation of spike number. A total of 762,116 full-length transcripts were generated, among which 214,500 were successfully annotated. We identified 185,424 unique genes, including 91,514 differentially expressed genes (DEGs). Functional enrichment analysis revealed 43 DEGs associated with the protein processing pathway in the endoplasmic reticulum. Notably, 8 DEGs were specifically expressed in young spikes and mature spikes of the contrasting spike-number germplasms, suggesting their potential role in spike number regulation. This study provides a high-quality transcriptomic resource and identifies candidate genes that may facilitate molecular breeding for enhanced seed yield in *A. mongolicum*.

## 1 Introduction

*Agropyron mongolicum* is a perennial forage grass native to arid and semi-arid desert regions, valued for its ecological resilience, nutritional quality, and economic importance (Fan et al., 2022). Adapted to drought and cold climates, the species is characterized by its early spring growth, robust root system, and palatable soft stems and leaves (Zhao et al., 2010; Zhang et al., 2019). These traits make *A. mongolicum* a crucial resource for ecological restoration and sustainable pasture systems (Che and Li, 2007; Han et al., 2017). However, its practical use is hindered by inherently low biomass, due to narrow leaves and limited tillering, and particularly by poor seed production, which restricts commercial propagation and large-scale forage development. The absence of comprehensive genomic information has further impeded progress in breeding and trait improvement. Thus, there is an urgent need to generate a valuable genetic resource and elucidate the molecular regulatory mechanisms associated with seed yield in *A. mongolicum*.

Recent advances in molecular biology have facilitated the exploration of protein processing mechanisms in the endoplasmic reticulum (ER), revealing their pivotal roles in plant growth and development. The ER protein processing pathway is known to mediate post-translational modifications such as glycosylation, hydroxylation, acylation, and disulfide bond formation. Among these, glycosylation is essential for protein stability and proper folding, which in turn supports cellular function and developmental processes. For instance, in rice (*Oryza sativa*), the gene *OsDNAJ15* encodes a heat shock protein 40 (HSP40) involved in ER-associated protein processing. Knockout of *OsDNAJ15* results in reduced plant height and increased leaf angle (Liu Y. et al., 2023). HSP40 family proteins are integral to protein folding, assembly/disassembly, and degradation. Additionally, *OsHSP40* mutants display significant changes in plant height and panicle length, leading to altered leaf morphology compared to the wild-type (Sun et al., 2016). These findings underscore the importance of ER-mediated protein processing in plant development, suggesting that similar mechanisms may be involved in regulating spike formation and yield traits in *A. mongolicum*.

Full-length transcriptome sequencing technologies have significantly advanced plant research due to their capacity for generating long reads that preserve full-length transcript structures. For instance, in *Zea mays*, Iso-Seq technology was employed to analyze the transcriptome architecture across six tissues (root, pollen, embryo, endosperm, young spikes, and young tassels) from the inbred line B73. This approach yielded 111,151 high-quality transcripts, representing approximately 70% of the annotated genes in the *Z. mays* RefGen_v3 genome. Notably, 57% of these transcripts were previously uncharacterized, including tissue-specific isoforms of known genes, while an additional 3% originated from entirely novel, unannotated gene loci. These findings underscored the complexity and dynamic nature of gene expression regulation in *Z. mays* (Wang et al., 2016). Similarly, Iso-Seq technology significantly improved gene annotation in wheat (*Triticum aestivum*), leading to the discovery of 3,026 novel genes and 9,591 new homologous isoforms. Furthermore, full-length transcripts were identified for 72 distinct wheat gluten protein genes. When integrated with second-generation RNA-seq data spanning four stages of grain development, transcriptomic analysis revealed 6,030 genes exhibiting isoform-specific expression patterns across developmental stages (Dong et al., 2015). In *Trifolium pratense* L. (red clover), researchers applied a hybrid approach combining PacBio Iso-Seq and Illumina RNA-seq to generate a comprehensive full-length transcriptome from roots, stems, leaves, and flowers. This strategy enabled the accurate annotation of key genes within the flavonoid biosynthesis pathway, yielding high-confidence gene models and identifying candidate genes relevant to biosynthesis and metabolic regulation. As a result, this work substantially advanced red clover transcriptome resources and facilitated the acceleration of molecular breeding efforts (Shi et al., 2021). Moreover, the use of single-cell full-length transcriptome technology—such as Smart-seq3—in *Arabidopsis thaliana* root tip cells has enabled the construction of the first cell type-specific alternative splicing atlas. This breakthrough revealed the spatiotemporal specificity of alternative splicing events during tissue differentiation and developmental progression (Shulse et al., 2019). Full-length transcriptome sequencing has also been successfully applied to various economically important crops, including cotton (*Gossypium* spp.) (Wang et al., 2018), sugar beet (*Beta vulgaris*) (Minoche et al., 2015), and alfalfa (*Medicago sativa* L.) (Zhang, 2018), where it has provided critical insights into gene structure and functional genomics. Collectively, these studies demonstrate the unique utility of full-length transcriptome technology for accurate gene annotation, alternative splicing analysis, and functional gene discovery, offering precise molecular targets for trait improvement in crops.

Building upon these technological advancements, the present study addresses the lack of genomic resources in *A. mongolicum* by integrating third-generation sequencing (Iso-Seq) with second-generation sequencing (RNA-seq) to characterize the transcriptomes of multi-spike and few-spike germplasms. Through the construction of a high-quality full-length transcript dataset, this work offers the first systematic annotation of genes potentially involved in tillering regulation and seed development in *A. mongolicum*. Notable candidates include heat shock proteins (HSPs), transcription factors (TFs), and components of key hormone signaling pathways. The resulting dataset provides a valuable transcriptomic resource and candidate genetic targets for the molecular enhancement of *A. mongolicum*, thereby laying the foundation for elucidating the genetic regulatory mechanisms underlying high seed yield.

## 2 Results

### 2.1 PacBio SMRT sequencing data

**Figure 1:**
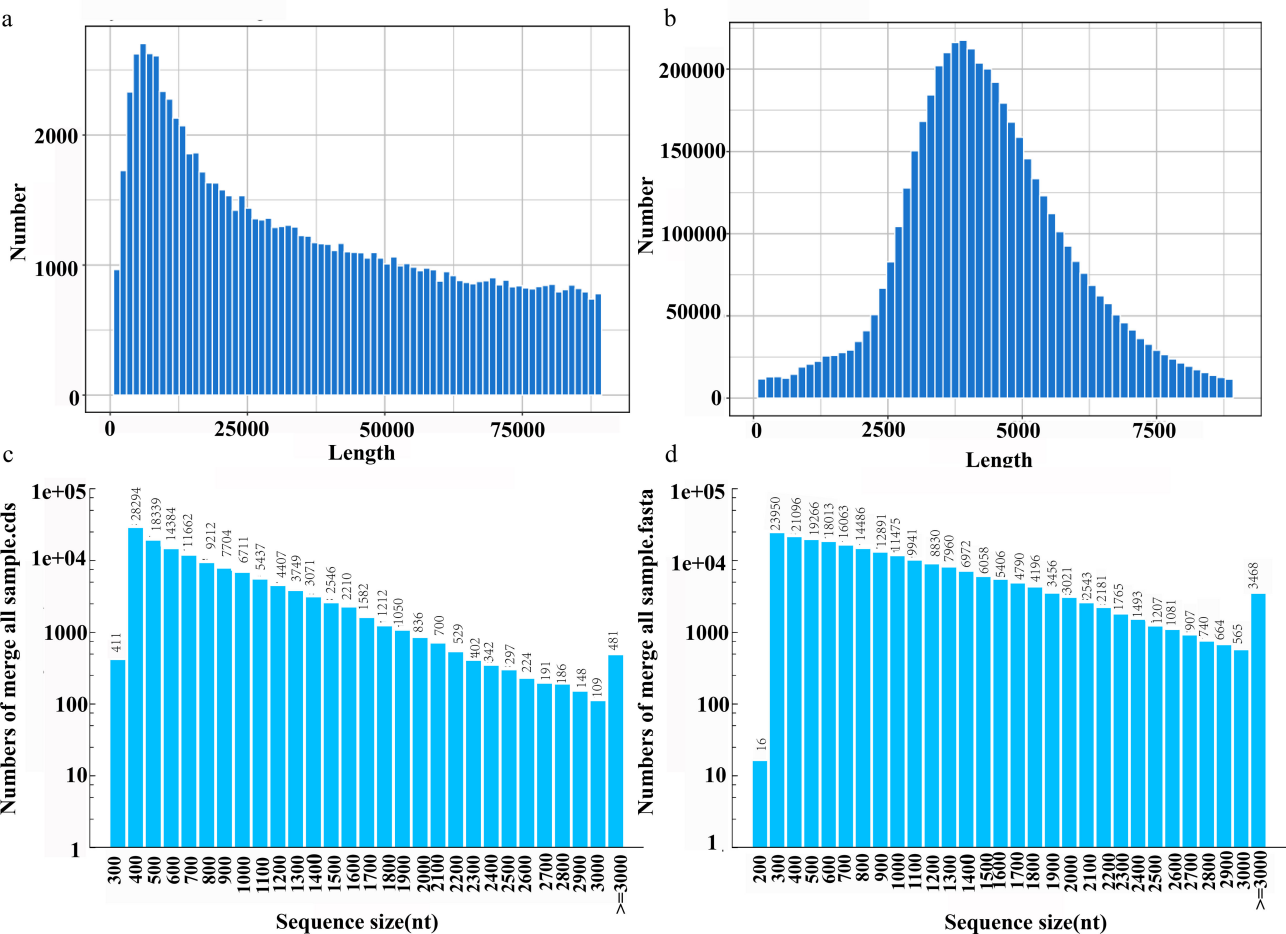
Length distribution of full-length transcriptome SMRT sequencing of *Agropyron mongolicum*. **(a)** Polymerase Reads Length. **(b)** SubReads Length. **(c)** Length distribution of merge all sample.fasta. **(d)** Number of CDSs in different length distributions.

A total of 0.2 million polymerase reads were generated, yielding 24.22 Gb of raw data. The reads exhibited exceptional continuity, with an average read length of 118.9 kb and an N50 value of 202.5 kb (**Figure 1a**). After stringent quality filtering, 5.02 million high-quality subreads were retained, totaling 23.86 Gb of data. Despite some shortening inherent to the error-correction process, these subreads maintained a high continuity (N50 = 4,985 bp) (**Figure 1b**).

Subsequently, subreads originating from the same circular molecule were integrated to produce circular consensus sequences (CCSs), resulting in 172,612 CCSs with an average length of 5,063 bp and a mean quality score of 0.99. Based on the presence of 5’-UTR, 3’-UTR, and poly(A) tail features, CCSs were classified into chimeric, non-chimeric, full-length, and non-full-length categories. A total of 762,116 full-length non-chimeric (FLNC) sequences were identified, each exhibiting an average quality score of 0.99 and an average length of 837.2 bp (**Supplementary Table S1**). After removing low-quality and redundant sequences, 214,500 high-confidence consensus reads were obtained, achieving an average quality score of 0.99 and demonstrating ultra-high accuracy (**Supplementary Tables S2**, **S3**; **Figure 1c**).

### 2.2 Coding sequence prediction

Coding sequence (CDS) prediction was performed on the full-length transcripts, resulting in the identification of 126,926 CDSs. The total CDS length reached 97,565,778 bp, with an N50 value of 924 bp (**Supplementary Table S4**). Among these, 97,217 CDSs ranged from 100 to 1,000 bp, 26,100 CDSs ranged from 1,000 to 2,000 bp, 3,128 CDSs ranged from 2,000 to 3,000 bp, and 481 CDSs were longer than 3,000 bp, accounting for 76.6%, 20.6%, 2.5%, and 0.4% of the total, respectively (**Figure 1d**).

### 2.3 Functional annotation

**Figure 2:**
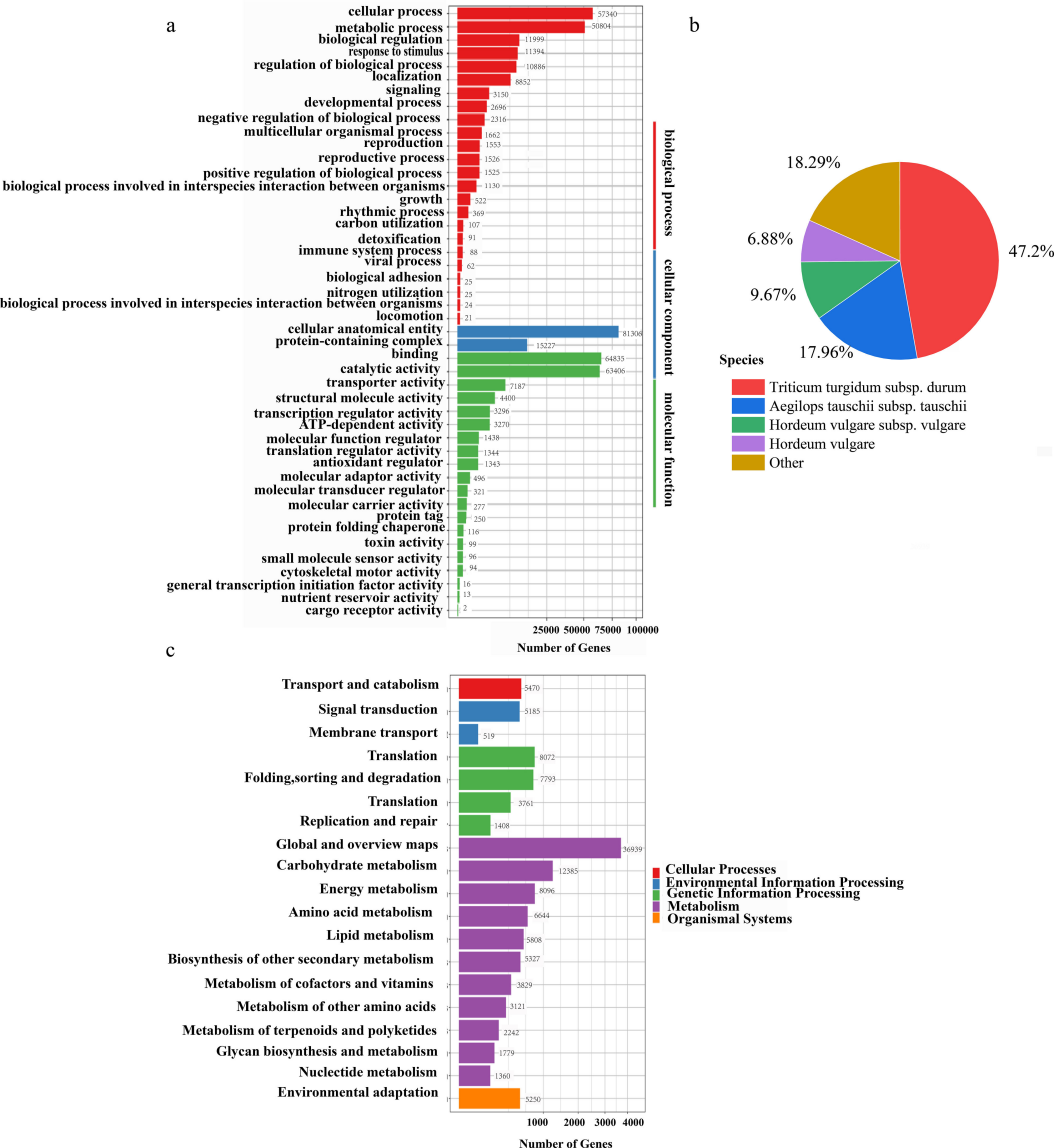
Functional annotation. **(a)** GO functional classification of transcripts. **(b)** The proportion of transcripts derived from homologous species and annotated by the NR database. **(c)** KEGG pathway classification.

A total of 214,500 transcripts were subjected to functional annotation across seven databases: NR, SwissProt, KEGG, KOG, Pfam, NT, and GO. Annotation results revealed 167,465 transcripts annotated in NR, 117,193 in SwissProt, 116,256 in KEGG, 91,589 in KOG, 108,431 in Pfam, 197,159 in NT, and 132,229 in GO databases. Notably, 99% of transcripts were successfully annotated in the NT database, reflecting the high conservation of nucleotide sequences, whereas KOG showed the lowest annotation rate (42.7%), indicating a relatively lower representation of conserved eukaryotic orthologs (**Supplementary Table S5**). NR-based homology analysis revealed that *Triticum turgidum* subsp. *durum* was the most predominant species, accounting for 47.20% of homologous annotations, followed by *Aegilops tauschii* (17.96%) and barley (*Hordeum vulgare* subspecies combined) at 16.55%. Remarkably, 18.29% of transcripts lacked species-specific homology, suggesting the presence of novel or highly divergent sequences (**Figure 2b**). KEGG pathway analysis categorized the annotated transcripts into five major functional groups: “cellular processes”, “environmental information processing” “metabolism”, “genetic information processing”, and “organismal systems”. Within these, “global and overview maps” under the “metabolism” category contained the highest number of transcripts (**Figure 2c**). The GO classification organized the transcripts into three domains: “biological processes”, “cellular components”, and “molecular functions” In the “biological processes” domain, the “cellular process” (57,340 transcripts) and “metabolic process” (50,804 transcripts) were the most prominent. In the “cellular components” domain, the majority of transcripts (81,306) were associated with “cellular anatomical entities” For “molecular functions” the dominant categories were “binding” (64,835 transcripts) and “catalytic activity” (63,406 transcripts) (**Figure 2a**).

### 2.4 Prediction of transcription factors

**Figure 3:**
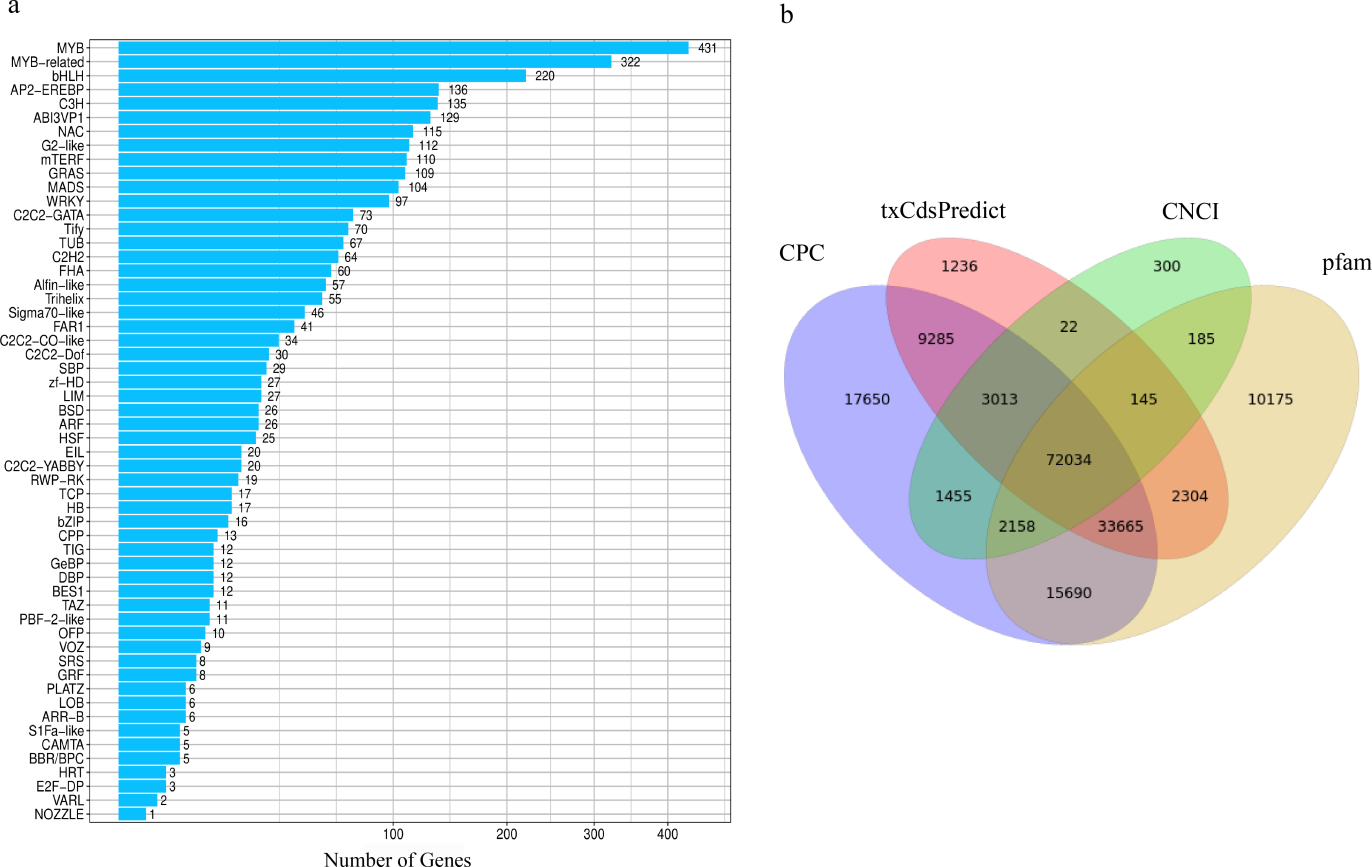
Prediction of TFs and lncRNAs prediction. **(a)** Transcription factor family classification. **(b)** Venn diagram of the lncRNAs numbers predicted by CPC, txCdsPredict, and CNCI software and the Pfam database. Numbers in the overlapping areas represent the number of shared lncRNAs.

This study systematically characterized TFs in *A. mongolicum*, revealing a complex regulatory framework potentially influencing yield-related traits and metabolic coordination. A total of 2,611 TFs were detected and classified into 56 families. Among them, the bHLH, MYB, and MYB-related families contained the highest numbers of predicted sequences (**Figure 3a**), highlighting their central roles in orchestrating spatiotemporal metabolic networks (Patra et al., 2013). This distribution mirrors conserved regulatory architectures observed in other high-yield crops, suggesting that these TF families have been evolutionarily optimized for resource allocation and developmental control.

### 2.5 Prediction of lncRNAs

To predict long non-coding RNAs (lncRNAs) in *A. mongolicum*, four computational approaches were utilized: CPC, txCdsPredict, CNCI, and Pfam database searches. These methods predicted 154,950, 121,704, 79,312, and 136,356 lncRNAs, respectively. Among these, 3,013, 145, 2,158, and 33,665 lncRNAs were exclusively predicted by three of the four methods, whereas 72,034 lncRNAs were consistently predicted by all four approaches (**Figure 3b**). Strikingly, the number of lncRNAs identified in *A. mongolicum* (~72,000) far exceeds that reported in the model plant *A. thaliana* (6,510) (Zhao et al., 2018). This substantial expansion suggests that *A. mongolicum* has evolved an extensive repertoire of non-coding regulatory elements, likely contributing to its complex mechanisms for stress adaptation and reproductive development.

### 2.6 Gene expression level analysis

**Figure 4:**
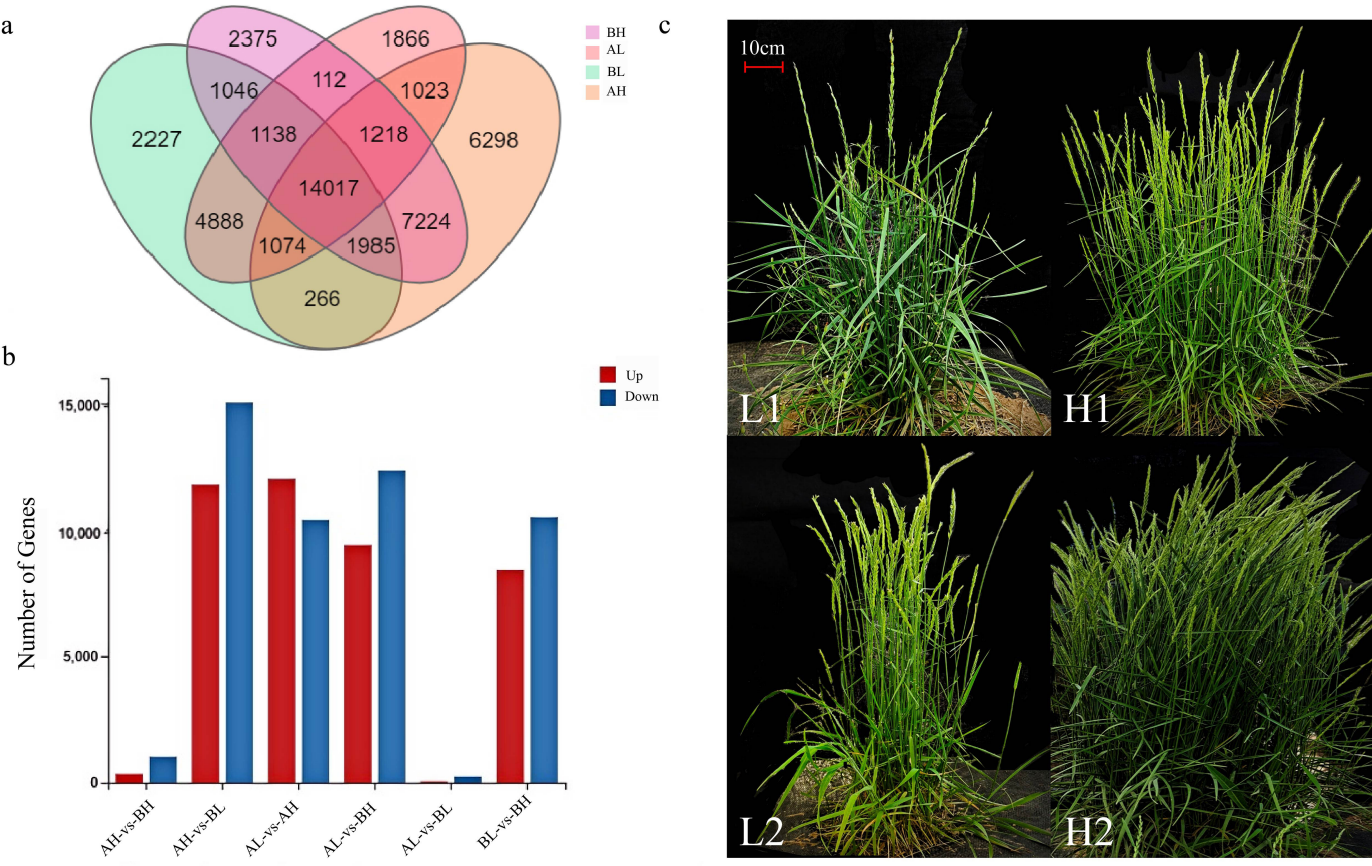
Gene Expression Level. **(a)** Statistics on the number of differentially expressed genes. The X-axis represents the difference comparison scheme for each group, and the Y-axis represents the corresponding number of DEGs. Red represents the number of upregulated DEGs, while blue represents the number of downregulated DEGs. **(b)** Expression level of unigenes in *Agropyron mongolicum.* **(c)** Phenotypic comparison of plants from different treatment groups (L: few-spike plants; H: multi-spike plants), scale bar = 10 cm.

Using an expression threshold of fragments per kilobase of transcript per million mapped reads (FPKM) >10, Venn diagrams were constructed to illustrate the distribution of unigenes across the four sample groups. A total of 14,017 unigenes were commonly expressed among all four groups (**Figure 4a**). The number of unique genes expressed in each group was 6,298 in AH, 1,866 in AL, 2,375 in BH, and 2,227 in BL, respectively.

Comparative transcriptomic analysis across different tissues of *A. mongolicum* collected at two distinct growth stages revealed significant transcriptional differences (**Figure 4c**). A total of 91,514 differentially expressed genes (DEGs) were identified across six pairwise comparisons. Among these, the comparisons of AH vs BL, AL vs AH, AL vs BH, and BL vs BH exhibited the highest numbers of DEGs, with 11,784, 12,010, 9,395, and 8,410 upregulated genes, and 15,029, 10,389, 12,338, and 10,495 downregulated genes, respectively (**Supplementary Table S6**). In contrast, the comparisons between AH vs BH and AL vs BL identified markedly fewer DEGs. These results indicate that transcriptomic differences are more pronounced between germplasms with contrasting spike numbers, whereas fewer changes occur between germplasms with similar spike numbers (**Figure 4b**).

### 2.7 Enrichment analysis of differentially expressed genes in KEGG pathways

**Figure 5:**
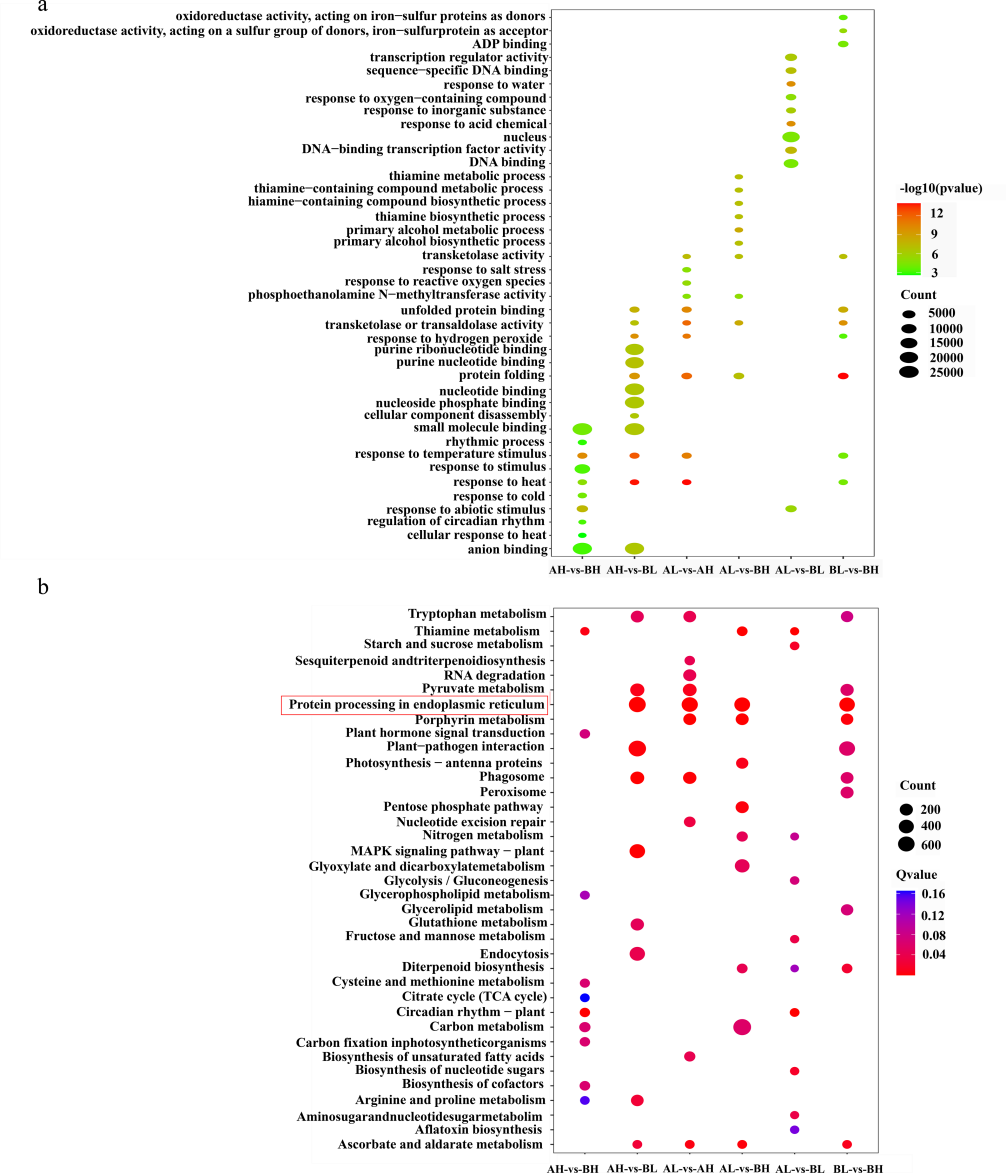
DEGs enrichment analysis. **(a)** DEGs GO enrichment bubble plot. **(b)** DEGs KEGG pathway enrichment bubble plot.

To gain insights into the biological functions of DEGs, KEGG pathway enrichment analysis was conducted. The ten pathways with the lowest Q-values were selected for detailed examination (**Figure 5b**). DEGs were significantly enriched in several pathways, most notably “protein processing in the endoplasmic reticulum” (ko04141), “porphyrin metabolism” (ko00860), and “phagosome” (ko04145) pathways. A smaller subset of DEGs was associated with the “aflatoxin biosynthesis” (ko00254) pathway, suggesting a potential but minor involvement.

### 2.8 GO enrichment analysis of differentially expressed genes

The GO enrichment analysis was performed on the set of 91,514 DEGs. Significant enrichment (Q-value < 0.001) was observed in various functional categories, particularly in “small molecule binding” (GO:0036094), “nucleotide binding” (GO:0000166), “nucleoside phosphate binding” (GO:1901265), “purine nucleotide binding” (GO:0017076), “purine ribonucleotide binding” (GO:0032555), and “nucleus” (GO:0005634) (**Figure 5a**). These enriched categories reflect the diverse functional roles of DEGs in cellular and molecular processes critical for growth and development.

### 2.9 Pathways related to protein processing in endothelial reticulum

KEGG annotation revealed that DEGs related to the “protein processing in the endoplasmic reticulum” (ko04141) pathway were significantly enriched in both the AL vs AH and BL vs BH comparison groups. Focusing on this pathway, the expression profiles of key genes were analyzed based on FPKM values, and heatmaps were generated to illustrate their dynamic expression patterns during spike development.

**Figure 6:**
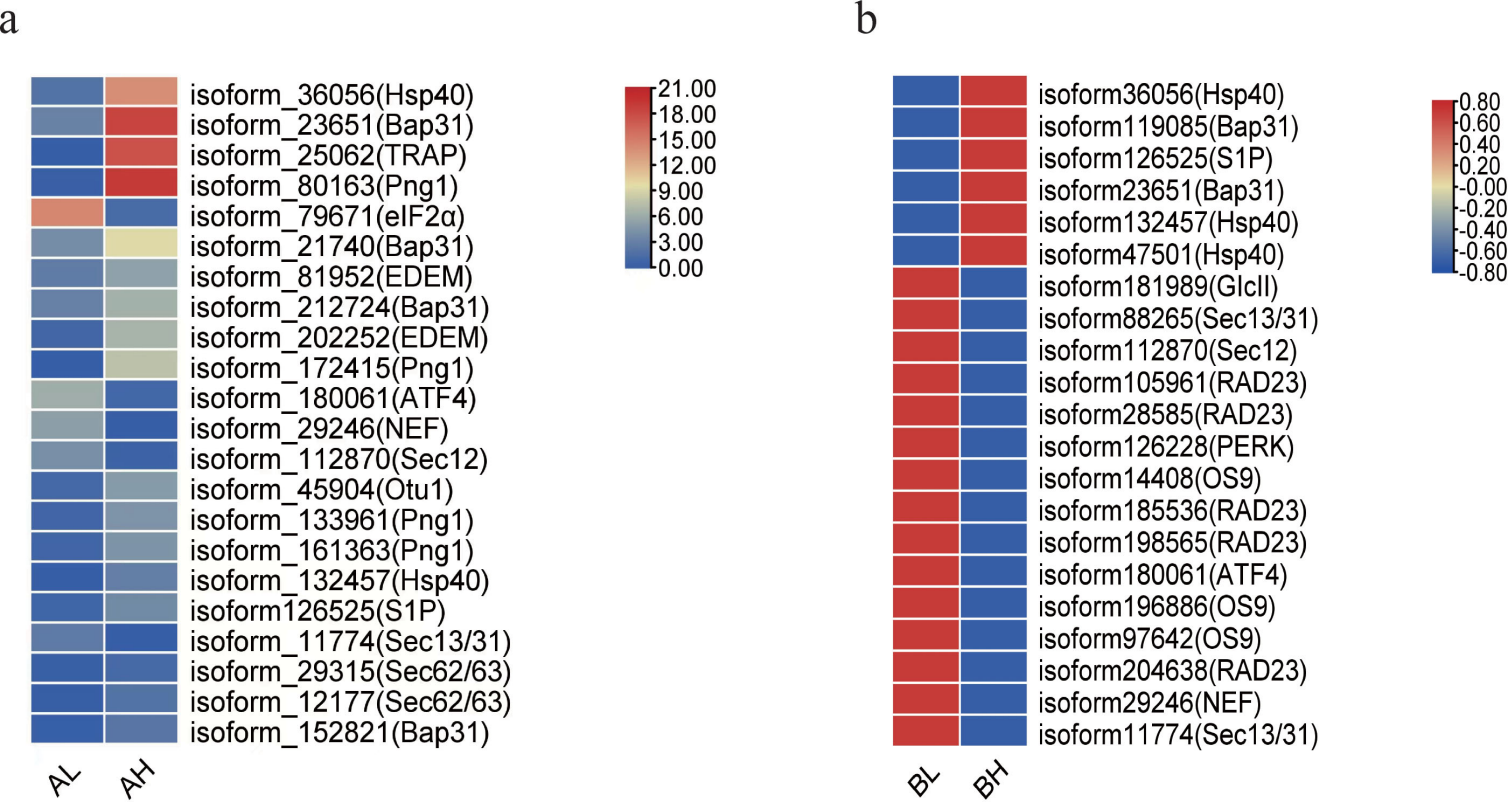
Heatmap of DEGs related to protein processing in the endoplasmic reticulum pathway in Agropyron mongolicum. **(a)** Heat map of differentially expressed genes related to protein processing in the endoplasmic reticulum pathway of Mongolian ice grass in AL vs AH group. **(b)** a) Heat map of differentially expressed genes related to protein processing in the endoplasmic reticulum pathway of Mongolian ice grass in BL vs BH group.

A total of 43 unigenes were mapped to the protein processing pathway. Expression analysis demonstrated distinct molecular regulatory patterns between multi-spike and few-spike plants at critical stages of spike development. During the booting stage (AL vs AH), genes encoding *Hsp40* (Heat Shock Protein), Bap31 (B cell receptor-associated protein 31), TRAP (tagged RNA affinity purification complex), and Png1 (peptide:N-glycanase 1)exhibited significantly higher expression levels in multi-spike plants compared to few-spike plants. Conversely, *eIF2α* (eukaryotic translation initiation factor 2α) was more highly expressed in few-spike plants, likely reflecting endoplasmic reticulum stress-mediated inhibition of protein synthesis. At the heading stage (BL vs BH), multi-spike plants sustained high expression of *Hsp40* and *Bap31*, while significantly downregulating critical components of the unfolded protein response (UPR), including PERK (protein kinase R-like endoplasmic reticulum kinase) and *ATF4* (activating transcription factor 4), as well as COPII vesicle transport-related genes such as *Sec12* and *Sec31* (Sec family proteins) (**Figure 6**). This regulatory balance suggests that multi-spike plants mitigate ER stress while promoting spike development, providing promising molecular targets for future genetic improvement of seed yield.

### 2.10 Verification by quantitative real-time polymerase chain reaction

**Figure 7:**
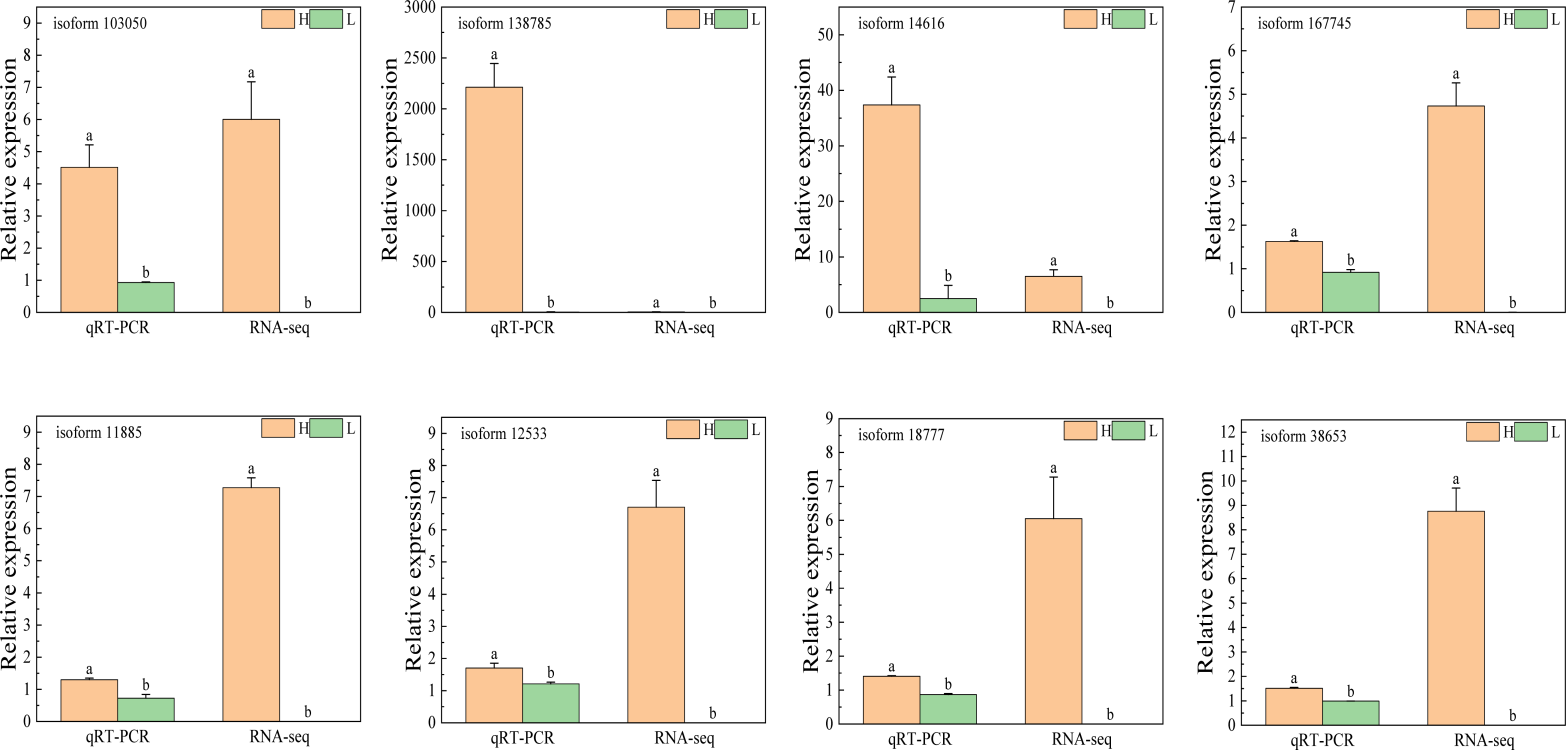
qRT-PCR validation of selected genes from RNA-seq.

To validate the reliability of the RNA-seq data, eight genes—*isoform12533*, *isoform103050*, *isoform11885*, *isoform14616*, *isoform18777*, *isoform38653*, *isoform138785*, and *isoform167745*—were randomly selected for quantitative real-time PCR (qRT-PCR) analysis. The qRT-PCR results were fully consistent with the RNA-seq data, confirming that these genes exhibited significantly higher expression levels in multi-spike plants compared to few-spike plants. These findings reinforce the accuracy and robustness of the transcriptomic analysis (**Figure 7**; **Supplementary Table S11**).

## 3 Discussion

### 3.1 Transcriptome sequencing

Compared to previous transcriptomic studies on this species, our findings provide a broader and more comprehensive transcriptomic resource, offering significant advantages in both technical application and biological discovery (Pan et al., 2016; Cechin et al., 2020). First, the full-length transcriptome generated in this study serves as a valuable reference for future genome annotation and assembly efforts in *A. mongolicum* and related species. Our results demonstrate that PacBio SMRT sequencing is particularly effective in capturing long transcripts. Compared to the reference transcriptomes of model plants such as *A. thaliana* and *O. sativa* (Chow et al., 2024), a greater number of transcripts exceeding 4,000 bp in length were identified in *A. mongolicum*, reflecting the strength of PacBio SMRT technology in generating extended and intact sequences. This may be partly attributed to the high proportion of long transcripts present in both the subread and full-length non-chimeric (FLNC) datasets (Ruan et al., 2022). In this study, we successfully obtained 762,116 accurate and high-quality full-length sequences, among which 214,500 transcripts were functionally annotated, providing invaluable data for gene structure analysis and functional genomics. Similar advantages of PacBio SMRT sequencing have been demonstrated in studies of other species. For example, in *Glycyrrhiza uralensis*, high-quality full-length transcriptomes were generated, facilitating the successful annotation of a large number of functional genes (Yu M. et al., 2021). Likewise, in *M. sativa*, PacBio SMRT sequencing provided comprehensive full-length transcript data supporting gene functional studies (Shang et al., 2021).

At the gene expression level, we identified 91,514 DEGs, a number significantly higher than those reported for the halophyte *Salicornia europaea* (Lou et al., 2024). The DEGs were primarily concentrated in four comparison groups: AH vs BL, AL vs AH, AL vs BH, and BL vs BH, underscoring the complex regulatory networks associated with spike number differentiation in *A. mongolicum*. Collectively, these data establish a solid foundation for future investigations into gene function, regulatory networks, and yield trait improvement in *A. mongolicum*.

### 3.2 Potential roles of transcription factors and lncRNAs

The TFs are key regulators of gene expression, mediating diverse physiological processes and plant responses to environmental stimuli. In this study, analysis of 214,500 full-length transcript sequences led to the identification of 3,046 TFs belonging to 56 different families. Notably, the MYB and bHLH families were the most abundant. The MYB family, one of the largest TF families in plants, plays central roles in growth and development, metabolic regulation, and responses to both biotic and abiotic stresses (Mou et al., 2023). MYB TFs are widely implicated in adaptive responses to nutrient stress, particularly low phosphorus availability, which otherwise limits plant growth and crop yield. MYB TFs regulate phosphorus transporter gene expression, enhancing tolerance to phosphorus-deficient conditions (Hu et al., 2024). Beyond nutrient stress, MYB TFs are crucial in responses to drought, salt stress, cold, and pathogen attacks. The R2R3-MYB subfamily, in particular, plays pivotal roles in regulating disease resistance and abiotic stress adaptation (Guan et al., 2024).

Although the role of MYB TFs in stress responses has been extensively studied, their potential function in regulating spike number remains underexplored. Emerging evidence suggests that MYB TFs influence multiple growth traits, including plant architecture, leaf morphology, and seed size (Wu X. et al., 2024). For example, the copper-induced MYB transcription factor *OsMYB67* regulates heading time and yield formation in rice. Knockout mutants of *OsMYB67* display an early heading phenotype and increased effective spike and grain numbers compared to the wild type, while overexpression delays heading (Ding et al., 2025). Moreover, *MFS2* (*MULTI-FLORET SPIKELET2*) in rice, an R2R3-MYB repressor-type transcription factor, forms a complex with TOPLESS/TOPLESS-associated proteins to inhibit floral organ identity gene expression, maintaining floret meristem determinacy. Loss of *MFS2* function leads to increased floral organ number and double-floret spikelet formation, highlighting its role in regulating spikelet number through meristem activity suppression (Li et al., 2020). In wheat, *TaMYB72* has been identified as a positive regulator of grain yield traits. Deletion mutants of *TaMYB72* show increased spike length and grain number per spike, although accompanied by delayed heading (Wu LF. et al., 2024). Similarly, *TabHLH27*, a bHLH family TF, is highly expressed during key stages of spike development in wheat, including the double-ridge stage, and likely influences spikelet number by regulating genes involved in inflorescence development (Wang et al., 2024). These findings underscore the crucial involvement of MYB and bHLH transcription factors in regulating spike-related traits and offer new avenues for targeted crop improvement.

lncRNAs (long non-coding RNAs)also play essential roles in plant development and yield trait regulation. In rice, lncRNAs are involved in controlling spikelet type and grain size, suggesting that they may influence spike number as well (Zhang et al., 2020). Studies in maize have demonstrated that lncRNAs can regulate spike development by interacting with protein-coding genes or participating in chromatin remodeling (Sun et al., 2020). Additionally, lncRNAs may function as distal regulatory elements, modulating the expression of spike-related genes across genomic regions (Parvathaneni et al., 2020). Although several lncRNAs associated with spike number regulation have been identified, comprehensive understanding of their regulatory mechanisms remains limited. Future research should focus on elucidating the molecular pathways by which lncRNAs influence spikelet formation, meristem activity, and yield traits, thereby expanding the potential for harnessing lncRNAs in breeding programs targeting high-yield phenotypes.

### 3.3 Gene families

Heat shock proteins (HSPs) play critical roles in enabling plant cells to adapt to abiotic stress, preventing protein denaturation and maintaining normal physiological functions. The complex structure of the small heat shock protein *Hsp21* and its natural substrate has opened new avenues for improving heat tolerance and potentially increasing spike number in crop species (Yu C. et al., 2021). Other major classes of HSPs—including HSP100 (Clp), HSP90, HSP70 (DnaK), HSP60, HSP40 (DnaJ), and small heat shock proteins (sHSPs)—also make substantial contributions to plant growth and stress tolerance (Sun et al., 2012). In this study, we found that *Hsp40* was significantly more highly expressed in multi-spike plants compared to few-spike plants, emphasizing its potential importance in spike development and overall plant vigor. Functional characterization of *OsDNAJ15*, a *Hsp40*/*DNAJ* homolog in rice, revealed that this gene positively regulates the DNA-binding activity of the spike number-related gene *OsMYB106* through interaction with *OsBAG4*, thereby influencing spike number formation (Liu Y. et al., 2023). The Hsp40 proteins may regulate downstream signaling pathways by cooperating with chaperone complexes. For instance, *OsHSP40* mutants exhibit marked phenotypic differences compared to wild-type rice, including altered plant height and spike length, likely due to effects on leaf vein formation, cell size, and cell number (Sun et al., 2016). Beyond signaling and gene expression regulation, *Hsp40* may also indirectly affect spike development by promoting the proper folding and assembly of proteins involved in cell division and differentiation. Studies in HeLa cells have shown that *Hsp40* collaborates with *Hsp70* to repair and refold nuclear proteins, demonstrating conserved functions in proteostasis (Hattori et al., 1993). Moreover, natural variation in *Hsp40* could serve as a potential mechanism for spike number diversity. SNP analysis of *LM7*, encoding the rice heat shock protein *OsHSP40*, identified a functional C-to-T SNP located in the coding region. Rice lines harboring this SNP exhibited improved agronomic traits, including increased flag leaf width, plant height, spike length, grain length, and grain weight (Wang et al., 2022). Although accumulating evidence suggests multiple mechanisms by which Hsp40 may regulate spike number, further studies are necessary to fully elucidate its specific modes of action.

Protein kinase R-like endoplasmic reticulum kinase (PERK) represents another important stress response factor. While the PERK signaling pathway in plants remains incompletely characterized, studies suggest that PERK regulates plant growth and development by modulating the ER stress response and autophagy. Activation of PERK signaling triggers downstream effectors such as *ATF4*, regulating the expression of genes involved in metabolism and stress adaptation (Liu F. et al., 2023). In *A. thaliana*, members of the PERK family have been implicated in abscisic acid (ABA) signaling and root growth regulation, highlighting their potential importance in plant development (Bai et al., 2009). In rice, expression profiling of *OsPERK* has revealed tissue-specific expression patterns under diverse stress conditions, providing valuable insights into its role during different developmental stages and environmental responses (Kesawat et al., 2023). Although direct evidence linking PERK to spike number regulation remains lacking, the involvement of PERK in ER stress signaling and cellular metabolism suggests that it may influence developmental processes related to tiller and spike formation. Notably, a gene associated with spike grain number, *RGN1*, has been identified in rice and appears to regulate tiller structure, indirectly affecting spike number (Li et al., 2022). Therefore, the potential role of PERK in spike development warrants further investigation. Currently, research on nucleotide exchange factors in plants is relatively limited. However, their potential roles in cytoskeleton assembly and developmental regulation remain an intriguing area for future studies. Of particular interest, NEF expression was detected exclusively in few-spike plants in this study, and not in multi-spike plants, suggesting a possible negative regulatory influence on spike number. Future research should prioritize elucidating the specific molecular functions of NEFs in plant development and their potential impact on reproductive traits.

## 4 Materials and methods

### 4.1 Plant materials

The plant materials used in this study consisted of multi-spike and few-spike germplasms of the cultivated variety “Mengnong No. 1” (*A. mongolicum*), developed by Inner Mongolia Agricultural University. Plants were cultivated in 2021 at the university’s experimental base (40°48′N, 111°41′E; elevation 1063 m; average annual precipitation 350 mm), with three biological replicates per germplasm. Multi-spike plants exhibited tall and uniform stature, strong tillering ability, rapid growth rate, and a high seed set rate, whereas few-spike plants were characterized by relatively shorter stature, reduced leaf mass, weaker tillering ability, and lower seed set rate.

### 4.2 Sampling

Leaves, stems, young spikes, and mature spikes from multi-spike and few-spike plants (*A. mongolicum*) were sampled at two developmental stages: the booting stage (A) and the heading stage (B) (**Supplementary Table S7**). Sampling was conducted in May 2023 at the experimental base of Inner Mongolia Agricultural University. For each growth stage, three multi-spike and three few-spike plants were randomly selected, with three biological replicates per condition, resulting in a total of 36 samples. Sample grouping details are provided in Appendices S8 and S9. Immediately after collection, samples were placed in cryogenic tubes, rapidly frozen in liquid nitrogen, and stored at –80°C for subsequent analysis.

### 4.3 RNA extraction and sequencing

Total RNA was extracted from the 36 samples. Purified mRNA was pooled for cDNA library construction, and sequencing was performed on the PacBio Sequel platform. RNA extraction, library construction, and sequencing were conducted by the BGI Research Center, Beijing. Following sequencing, the SMRT Analysis Suite was used for insertion fragment recognition (ROI) and reads classification, and the Quiver algorithm was employed to cluster and correct reads (Chin et al., 2013), thereby generating high-quality full-length consensus sequences. Full-length sequences from each library were merged for redundancy elimination and isoform expression quantification. Subsequent steps included transcript annotation using public databases and CDS prediction using TransDecoder. Differential expression and functional annotation analyses were performed based on isoform quantification results.

### 4.4 Functional annotation

Gene annotation was conducted using several public databases, including the NCBI nonredundant protein database (NR), the NCBI nonredundant nucleotide database (NT), SwissProt, GO, the Kyoto Encyclopedia of Genes and Genomes (KEGG), and the Eukaryotic Orthologous Groups (KOG) database. Sequence alignments were performed using the BLASTX algorithm (version 2.2.23) (Altschul et al., 1990).

### 4.5 CDS prediction

Coding sequence prediction was carried out using TransDecoder (version 3.0.1). The longest open reading frame (ORF) from each isoform was identified, followed by homologous sequence searches against the SwissProt database using BLASTX (version 2.2.23) to predict candidate coding regions.

### 4.6 Isoform expression quantification

Full-length transcripts obtained from PacBio Iso-Seq were clustered to determine isoform expression copy numbers. The number of full-length transcripts contained within each cluster was used as the original expression value. Expression levels were subsequently normalized across samples using the percentage normalization method.

### 4.7 Long non-coding RNAs prediction

Prediction of lncRNAs was performed using CPC (Kong et al., 2007) (version:0.9-r2), txCdsPredict (http://hgdownload.soe.ucsc.edu/admin/jksrc.zip) and CNCI (Sun et al., 2013) (https://github.com/www-bioinfo-org/CNCI) software tools. Thresholds for distinguishing lncRNAs from mRNAs were set as follows: for CPC, transcripts with scores ≥0 were classified as mRNAs and those ≤0 as lncRNAs; for CNCI, transcripts with scores ≥0 were classified as mRNAs and those ≤0 as lncRNAs; and for txCdsPredict, transcripts with scores ≥500 were classified as mRNAs and those ≤500 as lncRNAs.

### 4.8 Expression analysis of the candidate genes by real-time qPCR

Total RNA was extracted using the Eastepfi Super Total RNA Extraction Kit (Promega, Beijing, China) according to the manufacturer’s instructions. First-strand cDNA was synthesized using the TransScript All-in-One First-Strand cDNA Synthesis SuperMix for qPCR. The qRT-PCR was carried out using the PerfectStart™ Green qPCR SuperMix on a LightCycler^®^ 480 II Real-Time PCR System. Specific primer sequences used for qRT-PCR are provided in **Supplementary Table S10**. *Actin* was employed as the internal control gene. Each sample was analyzed in three technical replicates, and relative gene expression levels were calculated using the 2^-ΔΔCT^ method (Schmittgen and Livak, 2008).

## 5 Conclusions

In this study, we generated high-quality full-length transcriptome data for *A. mongolicum*, obtaining a total of 762,116 full-length transcripts, of which 214,500 sequences were successfully annotated. We detected 185,424 genes, among which 91,514 exhibited differential expression. The GO and KEGG enrichment analyses of the DEGs revealed distinct molecular characteristics associated with plant growth and development at varying spike number levels, with a notable enrichment in the protein processing pathway of the endoplasmic reticulum. Further detailed analysis of this pathway suggested that *Hsp40*, *PERK*, *NEF*, and *Png1* may play critical roles in regulating biomass accumulation and spike development in *A. mongolicum*. The relative expression levels of eight DEGs were validated using qRT-PCR, confirming that these genes exhibited significantly higher expression in multi-spike plants compared to few-spike plants. Overall, the full-length transcriptome dataset obtained in this study demonstrates broad transcript coverage and high sequence integrity, providing a valuable resource for future functional genomic studies in *A. mongolicum*. These results lay a solid foundation for the molecular dissection of yield-related traits and open new avenues for the genetic improvement of this important forage species.

## Data Availability

The datasets presented in this study can be found in online repositories. The names of the repository/repositories and accession number(s) can be found below: https://www.ncbi.nlm.nih.gov/, PRJNA1195816(https://dataview.ncbi.nlm.nih.gov/object/PRJNA1195816?reviewer=krqrt041giut79e9pobteldkfc).
